# Supplementary figures and images for: Feral frogs, native newts, and chemical cues: identifying threats from and management opportunities for invasive African Clawed Frogs in Washington state
Source: PeerJ. 2024 May 10;12:e17307. doi: 10.7717/peerj.17307 (PMC11090105; doi:10.7717/peerj.17307)

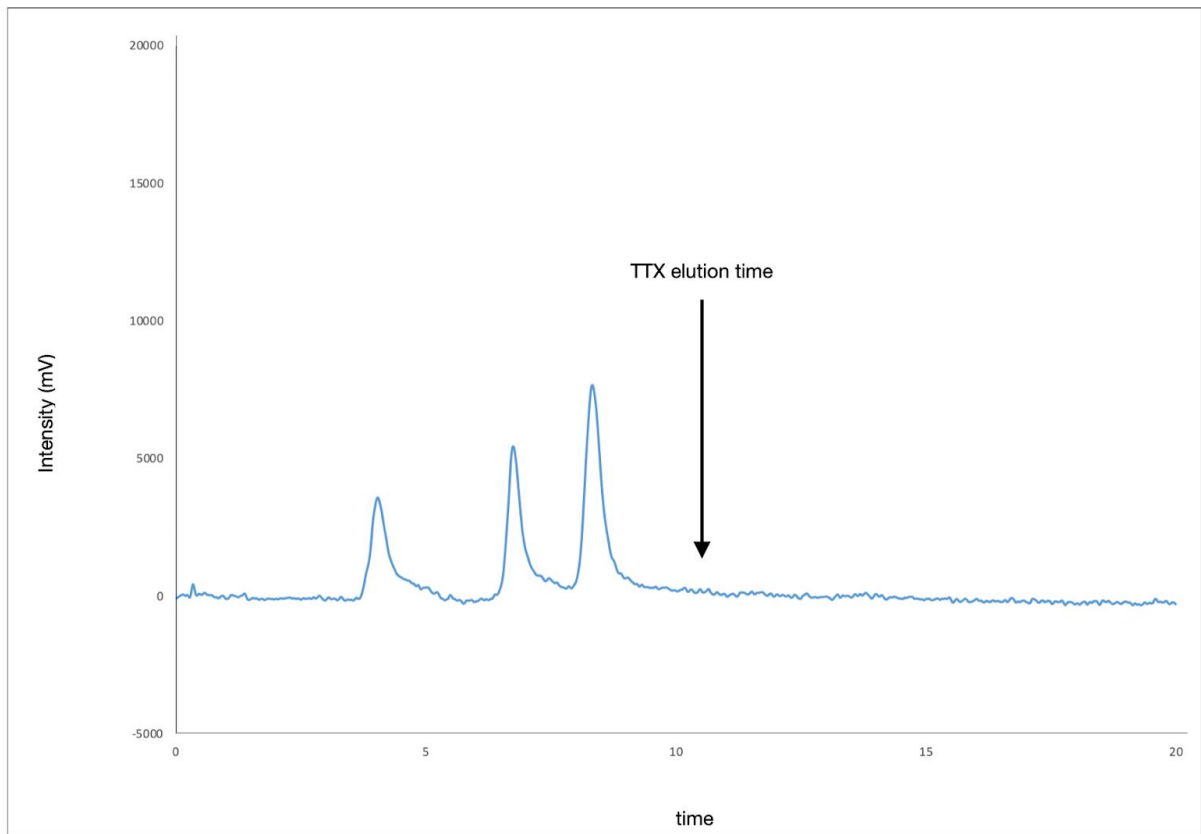

Supplement: Figure Appendix S1 [file peerj-12-17307-s001.pdf]
